# Configurations of Family Risk Factors and Mental Health Symptoms Among Grade 4–6 Children in Guangdong, China: A Large-Scale Crisp-Set Qualitative Comparative Analysis Based on a Cumulative Risk Model

**DOI:** 10.31083/AP45872

**Published:** 2025-08-18

**Authors:** Kaifeng Liu, En Fu, Ziyi Deng, Minrui Zhang, Yongxing Guo, Qiufeng Gao, Ruixiang Gao, Lei Mo

**Affiliations:** ^1^Intelligence and Command Center, Tianhe District Branch of Guangzhou Public Security Bureau, 510655 Guangzhou, Guangdong, China; ^2^Division of Behavioral Health Services and Policy Research, Vagelos College of Physicians and Surgeons, Columbia University, New York, NY 10032, USA; ^3^Center for Studies of Psychological Application, Guangdong Key Laboratory of Mental Health and Cognitive Science, and School of Psychology, South China Normal University, 510631 Guangzhou, Guangdong, China; ^4^Department of Pediatrics, Guangdong Provincial Hospital of Traditional Chinese Medicine, 510120 Guangzhou, Guangdong, China; ^5^DiggMind Psychometric Testing Technology Co., 510660 Guangzhou, Guangdong, China; ^6^Department of Psychology, School of Education Science, Hunan Normal University, 410081 Changsha, Hunan, China; ^7^Department of Sociology, School of Government, Shenzhen University, 518060 Shenzhen, Guangdong, China

**Keywords:** mental health, child, risk factor, family characteristics, qualitative research

## Abstract

**Background:**

Children’s mental health is significantly influenced by family environments, where multiple risks often coexist, exert unequal impacts, and combine in different configurations that can result in diverse developmental outcomes. This study examines how different configurations of cumulative family risks influence mental health symptoms in Chinese children using a novel person-centered approach.

**Materials and Methods:**

Data were collected through a large-scale, semester-based comprehensive survey of 34,041 children in Grades 4 to 6 in an economically underdeveloped county-level city in Guangdong, China, during November and December, 2022. Six family risk indicators were examined: incomplete family structure, parent–child separation, financial hardship, low parental education, lack of family intimacy, and family conflict. The Pediatric Symptom Checklist was used to measure children’s mental health outcomes, consisting of internalizing problems, externalizing problems, and attention problems. Crisp-set qualitative comparative analysis was applied to identify specific configurations of family risks associated with different mental health outcomes.

**Results:**

No single risk factor was found necessary or sufficient to explain mental health outcomes; configurations of multiple risks were more predictive. Externalizing and attention symptoms shared one configuration, which also contributed to internalizing symptoms. Additionally, three distinct configurations were uniquely associated with internalizing symptoms. Only lack of family intimacy and family conflict consistently appeared as detrimental across all configurations.

**Conclusions:**

This study reinforces the cumulative risk model and aligns with the concepts of multifinality and equifinality. It emphasizes the importance of monitoring children with coexisting risks and targeted interventions addressing one or two key factors rather than all factors simultaneously. Future research should adopt longitudinal designs and include broader factors.

## Main Points

∙ Examines how family risks configurations affect Chinese children’s mental health.

∙ Uses crisp-set qualitative comparative analysis.

∙ Supports the cumulative risk model and multifinality/equifinality concepts.

## 1. Introduction

Children’s development is associated with physiological, psychological, and social changes [1, 2] that can lead to various internal conflicts and pressures, placing them at high risk for mental health problems. Disrupted family structures, impoverished socioeconomic status, and disharmonious family atmosphere can also severely affect children’s mental health, potentially leading to psychiatric disorders such as depression, anxiety, conduct disorders, attention deficit issues, and even suicide [3, 4, 5, 6, 7]. Moreover, children often struggle to articulate their psychological distress and lack adequate support systems, which increases the likelihood of their mental health issues being overlooked and potentially developing into psychiatric disorders.

The problem of early mental health issues and the need for early intervention is particularly pressing in China, where rapid social and economic shifts are continually reshaping the environments in which children grow and develop [8]. As these transformations unfold, the family unit, traditionally a central pillar of Chinese society [9], is also experiencing changes. Shifts in family dynamics—such as evolving parenting styles, changes in family structures due to urban migration, and changing societal expectations—have far-reaching effects on children’s mental health [10]. Consequently, understanding how family risk factors influence children’s mental health in the context of contemporary China is vital for academic inquiry and plays a crucial role in informing intervention strategies designed to improve children’s overall well-being.

Family risks rarely occur in isolation as the presence of one risk factor, such as parental divorce, often correlates with other risk factors, such as family conflict. The cumulative risk model (CRM) suggests that exposure to multiple risk factors can have a greater negative impact on children’s development than exposure to individual factors alone [11, 12, 13, 14]. Additionally, different combinations of family risks can lead to distinct developmental outcomes, irrespective of the total number of risks [15, 16, 17]. However, traditional research has largely relied on correlation-based methods, such as regression analyses, which primarily examine the additive and independent effects of individual variables. These approaches are limited in capturing the complex interactions and combinations of risk factors, especially when multiple risks co-occur and interact simultaneously. Therefore, there remains a significant gap in understanding how different configurations or combinations of family risks uniquely contribute to children’s developmental outcomes.

To address this limitation, the present study employs qualitative comparative analysis (QCA), an emerging person-centered analytical method that differs from traditional regression analyses [18] and is increasingly applied in psychological and behavioral research [19]. While regression analyses focus on the effects of individual variables and often face challenges related to collinearity and limited interpretability of multiple interaction terms, QCA explicitly examines combinations of causal conditions. This method does not assume independence among variables, allowing for a comprehensive exploration of all possible configurations of family risk factors [20]. By using QCA, this study aims to clarify how specific combinations of risk factors contribute uniquely to developmental outcomes, providing nuanced insights that traditional methodologies may overlook.

We followed the key principles outlined by Li *et al*. [12], including systematicity, typicality, correlation, development, uniqueness, and feasibility (for detailed selection criteria, please refer to Chen *et al*. [21]) to identify six key risk factors across three family domains: family structure (parental divorce and parent–child separation), family resources (poverty and low parental education), and family atmosphere (low intimacy and high conflict). Although not exhaustive, these factors represent the most widely recognized risks identified in research, both in China [22] and internationally [15, 23]. Moreover, we followed the dichotomous coding practices established in previous CRM literature to ensure that only significant risks were considered [11, 12, 21, 24].

Regarding mental health indicators, we focused on three critical dimensions of children’s mental health—internalizing problems, externalizing problems, and attention problems—as identified by the well-established Pediatric Symptom Checklist (PSC) [25]. These indicators encompass a broad range of emotional, behavioral, and attentional difficulties that are prevalent among school-aged children and closely linked to significant short- and long-term impairments in academic and social functioning [26, 27, 28, 29, 30, 31]. Given the high prevalence and the significant short- and long-term consequences of these social, emotional, and behavioral problems among children, identifying the family risk configurations that contribute to them is of crucial importance.

## 2. Methods

### 2.1 Participants and Procedures

The data were collected using a semester-based comprehensive survey of student mental health status organized by the local education bureau in an economically underdeveloped county-level city in Guangdong, China, during November and December 2022. The survey involved all grade 4–6 students in the region and was administered via a self-developed online platform called DiggMind (version 2.0) (DiggMind Psychometric Testing Technology Co., Ltd., Guangzhou, Guangdong, China). A total of 43,494 students completed the survey in the school computer lab on a class-by-class basis, taking approximately 15 minutes. Proper procedures were followed to obtain permission to access and utilize the anonymous data for this study. After excluding the participants who failed the data consistency check (i.e., those with inconsistent self-reported information, responses deemed invalid), a total of 34,041 participants (validity rate 78.3%) were included in the analysis. Among them, 18,339 (53.8%) were boys and 15,702 (46.1%) were girls; 24,207 (71.1%) were from rural areas and 9834 (28.8%) from urban areas.

### 2.2 Measures

#### 2.2.1 Pediatric Symptom Checklist

The 35-item PSC, originally developed by Jellinek *et al*. [25], was used to measure emotional and behavioral problems among the student respondents. Although the PSC was initially designed as a parent-rating scale, it has also been validated as a self-report scale for older children and adolescents aged 9 to 18 [32]. The PSC comprises three dimensions: Internalizing Symptoms (17 items; e.g., “I often feel sad and unhappy”, Cronbach’s α = 0.90) reflect inward-directed emotional difficulties, such as anxiety, depressive moods, somatic complaints, and social withdrawal, frequently associated with psychiatric disorders like major depressive disorder and generalized anxiety disorder [33]. Externalizing Symptoms (11 items; e.g., “I often get into fights with other children”, Cronbach’s α = 0.88) pertain to outward-directed behaviors, including aggression and rule-breaking, commonly linked to conduct disorder and oppositional defiant disorder [34]. Attention Symptoms (7 items; e.g., “I like to move, and when I do, I can’t stop moving”, Cronbach’s α = 0.81) are characterized by hyperactivity, concentration difficulties, and impulsivity, central to attention-deficit/hyperactivity disorder (ADHD) [35]. All items were rated on a 3-point scale, 0 = Not True, 1 = Sometimes True, and 2 = Often True, and the mean scores for each dimension were calculated for analysis. To facilitate comprehension for younger children, each PSC item was accompanied by a picture illustration and a read-aloud function on our self-established survey platform DiggMind.

#### 2.2.2 Cumulative Family Risks Questionnaire

The cumulative family risks questionnaire includes six constructs and has been well validated by a number of previous studies [21, 24]. Incomplete Family Structure was measured using the question, “Who do you currently reside with?” Responses were coded as 1 (at risk) if participants did not select both “biological father” and “biological mother”, and 0 (no risk) otherwise. Parent–child Separation was assessed by the item, “In the past six months, I did not live with my parents because they worked outside”, with “Yes” coded as 1 and “No” coded as 0. Family Financial Hardship was measured using the 4-item Family Economic Pressure Scale (e.g., “My family does not have enough money to buy new clothes”, Cronbach’s α = 0.79) scored on a 5-point scale. Scores at or above the 75th percentile were coded as 1 (at risk) and those below as 0, based on established practices in CRM literature [11, 12, 21, 24]. For Poor Parental Education Level, the educational levels of both parents were assessed, with responses coded as 1 if both parents had an education level below “high school” and 0 if at least one parent exceeded this level. Lack of Family Intimacy was measured using 16 items (e.g., “Family members try their best to support one another during difficult times”, Cronbach’s α = 0.89) scored on a 5-point scale. Based on previous research guidelines [21, 24], scores at or below the 25th percentile were coded as 1 and those above as 0. Lastly, Family Conflict was assessed using 9 items (e.g., “There are frequent arguments in my family”, Cronbach’s α = 0.70) scored 1 for “No” and 2 for “Yes”. Based on established practices in CRM literature [11, 12, 21, 24], scores at or above the 75th percentile were coded as 1 and scores below as 0.

### 2.3 Data Analysis

The data analysis proceeded in two steps. First, we presented descriptive statistics, including means and standard deviations for the three symptom dimensions and their group differences according to the six family risk factors, gender, and urban–rural status. Second, we conducted the main analysis using crisp-set QCA via fsQCA v.4.1 software (Department of Sociology, University of California, Irvine, CA, USA) [36], as all independent variables were dichotomous.

We calibrated the data for crisp-set QCA by binarizing each symptom dimension using the 75th percentile as the threshold, consistent with established practices in CRM literature [11, 12, 21, 24] and QCA applications in large-N studies [37]; individuals scoring in the top 25% were coded as 1, indicating a high level of symptoms, while all others were coded as 0. We then assessed the necessity of each of the eight conditions (six family risks and two demographics) for each outcome. Conditions with raw consistency scores exceeding 0.80 [38] and coverage scores greater than 0.50 [39] were identified as necessary for each outcome. Subsequently, we constructed a truth table for each symptom dimension, displaying all possible combinations (“configurations”) of the causal conditions. Configurations were identified as sufficient if they had a frequency greater than 1 and a consistency score exceeding 0.80. In other words, if 80% or more of the cases with a specific configuration exhibited the outcome, membership of the outcome condition was assigned to that configuration. Here, we selected “0.80” as the membership threshold in the truth table for two reasons: on the one hand, “0.80” is the default threshold provided by the fsQCA software; on the other hand, there were substantial consistency gaps between configurations above and below this threshold across the three truth tables. Outcome membership for each configuration was then recoded as either 1 (full membership) or 0 (full non-membership) based on this threshold. Finally, we used the “standard analyses” function in fsQCA [36] to derive complex, parsimonious, and intermediate solutions for each outcome. When generating intermediate solutions, all eight conditions were explicitly specified as either “present” or “absent”.

## 3. Results

### 3.1 Descriptive Statistics

Table 1 presents the interquartile ranges and Spearman correlation coefficients of the nine variables studied. Table 2 presents the differences in these symptom dimensions according to family risk factors, gender, and urban–rural status using Mann-Whitney U tests.

**Table 1.** **Interquartile ranges and spearman correlations of study variables**.

	Q1	Q2	Q3	1	2	3	4	5	6	7	8	9
1. Incomplete Family Structure	0.000	0.000	1.000	1								
2. Parent–child Separation	0.000	0.000	1.000	0.259^*⁣**^	1							
3. Poor Parental Education Level	0.000	1.000	1.000	0.072^*⁣**^	0.056^*⁣**^	1						
4. Family Financial Hardship	0.000	0.000	1.000	0.044^*⁣**^	0.037^*⁣**^	0.109^*⁣**^	1					
5. Lack of Family Intimacy	0.000	0.000	1.000	0.116^*⁣**^	0.063^*⁣**^	0.045^*⁣**^	0.064^*⁣**^	1				
6. Family Conflict	0.000	0.000	1.000	0.052^*⁣**^	0.010	–0.003	0.078^*⁣**^	0.327^*⁣**^	1			
7. Internalizing Symptoms	0.059	0.235	0.471	0.071^*⁣**^	0.014^**^	0.028^*⁣**^	0.117^*⁣**^	0.251^*⁣**^	0.285^*⁣**^	1		
8. Externalizing Symptoms	0.000	0.091	0.273	0.062^*⁣**^	0.018^**^	0.026^*⁣**^	0.126^*⁣**^	0.258^*⁣**^	0.3168^*⁣**^	0.745^*⁣**^	1	
9. Attention Symptoms	0.000	0.286	0.714	0.057^*⁣**^	0.005	0.034^*⁣**^	0.143^*⁣**^	0.212^*⁣**^	0.270^*⁣**^	0.739^*⁣**^	0.726^*⁣**^	1

^**^p < 0.01, ^*⁣**^p < 0.001.

**Table 2.** **Median (Q1, Q3) of the symptom dimensions by family risk factors, gender, and urban–rural status**.

Incomplete Family Structure		No	Yes	*Z*
		(n = 22,055, 64.79%)	(n = 11,986, 35.21%)	
	Internalizing Symptoms	0.177 (0.059, 0.471)	0.235 (0.118, 0.529)	–13.071^*⁣**^
	Externalizing Symptoms	0.091 (0.000, 0.273)	0.091 (0.000, 0.273)	–11.481^*⁣**^
	Attention Symptoms	0.286 (0.000, 0.571)	0.429 (0.143, 0.714)	–10.463^*⁣**^
Parent–child Separation		No	Yes	*Z*
		(n = 24,499, 71.97%)	(n = 9542, 28.03%)	
	Internalizing Symptoms	0.235 (0.059, 0.471)	0.235 (0.0589, 0.529)	–2.661^**^
	Externalizing Symptoms	0.091 (0.000, 0.273)	0.091 (0.000, 0.273)	–3.351^**^
	Attention Symptoms	0.286 (0.000, 0.714)	0.286 (0.000, 0.714)	–0.936
Poor Parental Education Level		No	Yes	*Z*
		(n = 14,209, 41.74%)	(n = 19,832, 58.26%)	
	Internalizing Symptoms	0.235 (0.059, 0.471)	0.235 (0.059, 0.471)	–5.217^*⁣**^
	Externalizing Symptoms	0.091 (0.000, 0.273)	0.091 (0.000, 0.273)	–4.764^*⁣**^
	Attention Symptoms	0.286 (0.000, 0.571)	0.286 (0.000, 0.714)	–6.362^*⁣**^
Family Financial Hardship		No	Yes	*Z*
		(n = 23,610, 69.36%)	(n = 10,431, 30.64%)	
	Internalizing Symptoms	0.177 (0.059, 0.471)	0.294 (0.118, 0.529)	–21.576^*⁣**^
	Externalizing Symptoms	0.091 (0.000, 0.273)	0.091 (0.000, 0.364)	–23.265^*⁣**^
	Attention Symptoms	0.286 (0.000, 0.571)	0.429 (0.143, 0.714)	–26.319^*⁣**^
Lack of Family Intimacy		No	Yes	*Z*
		(n = 24,761, 72.74%)	(n = 9280, 27.26%)	
	Internalizing Symptoms	0.177 (0.059, 0.353)	0.412 (0.177, 0.706)	–46.271^*⁣**^
	Externalizing Symptoms	0.091 (0.000, 0.182)	0.182 (0.000, 0.455)	–47.515^*⁣**^
	Attention Symptoms	0.286 (0.000, 0.571)	0.571 (0.143, 0.857)	–39.182^*⁣**^
Family Conflict		No	Yes	*Z*
		(n = 23,910, 70.24%)	(n = 10,131, 29.76%)	
	Internalizing Symptoms	0.177 (0.059, 0.353)	0.412 (0.177, 0.706)	–52.663^*⁣**^
	Externalizing Symptoms	0.091 (0.000, 0.182)	0.182 (0.000, 0.455)	–58.362^*⁣**^
	Attention Symptoms	0.286 (0.000, 0.571)	0.571 (0.143, 0.857)	–49.78^*⁣**^
Gender		Male	Female	*Z*
		(n = 18,339, 53.87%)	(n = 15,702, 46.13%)	
	Internalizing Symptoms	0.235 (0.059, 0.471)	0.235 (0.059, 0.471)	–4.835^*⁣**^
	Externalizing Symptoms	0.091 (0.000, 0.273)	0.091 (0.000, 0.273)	–11.825^*⁣**^
	Attention Symptoms	0.286 (0.000, 0.714)	0.286 (0.000, 0.571)	–10.371^*⁣**^
Area		Rural	Urban	*Z*
		(n = 24,207, 71.11%)	(n = 9834, 28.89%)	
	Internalizing Symptoms	0.177 (0.059, 0.471)	0.235 (0.118, 0.529)	–15.621^*⁣**^
	Externalizing Symptoms	0.091 (0.000, 0.273)	0.091 (0.000, 0.273)	–10.295^*⁣**^
	Attention Symptoms	0.286 (0.000, 0.571)	0.429 (0.143, 0.714)	–14.996^*⁣**^

^**^p < 0.01, ^*⁣**^p < 0.001.

### 3.2 Findings of the Crisp-Set Qualitative Comparative Analysis

Data from 34,041 children were analyzed. First, we analyzed the necessary conditions for the presence and absence of the outcomes by grouping cases that shared the same combinations of causal conditions, creating a truth table. As shown in Table 3, none of the hypothesized causal conditions exceeded the consistency score threshold of 0.8 [38] and the coverage score threshold of 0.5 [39], indicating that no condition was necessary for the outcomes to occur.

**Table 3.** **Analysis of necessary conditions**.

Outcome: Internalizing Symptoms	Present		Absent	
	Consistency	Coverage	Consistency	Coverage
Incomplete Family Structure	0.400022	0.307776	0.334300	0.692224
Parent–child Separation	0.300043	0.289981	0.272976	0.710019
Poor Parental Education Level	0.590111	0.274405	0.579798	0.725595
Family Financial Hardship	0.355780	0.314543	0.288086	0.685457
Lack of Family Intimacy	0.458903	0.456034	0.203393	0.543966
Family Conflict	0.506723	0.461258	0.219912	0.538742
Gender	0.481024	0.282512	0.45926	0.717488
Area	0.333008	0.312284	0.272493	0.687716
∼Incomplete Family Structure	0.599978	0.250873	0.665700	0.749127
∼Parent–child Separation	0.699957	0.263480	0.727024	0.736520
∼Poor Parental Educational Level	0.409889	0.266029	0.420202	0.733971
∼Family Financial Hardship	0.644220	0.251631	0.711914	0.748369
∼Lack of Family Intimacy	0.541097	0.201527	0.796607	0.798473
∼Family Conflict	0.493277	0.190255	0.780088	0.809745
∼Gender	0.518976	0.260974	0.546074	0.739026
∼Area	0.666992	0.254100	0.727507	0.745900
Outcome: Externalizing Symptoms	Presence		Absence	
	Consistency	Coverage	Consistency	Coverage
Incomplete Family Structure	0.394950	0.240113	0.340435	0.759887
Parent–child Separation	0.299712	0.228883	0.275024	0.771117
Poor Parental Education Level	0.596130	0.219040	0.578904	0.780960
Family Financial Hardship	0.382874	0.267472	0.285602	0.732528
Lack of Family Intimacy	0.473309	0.371659	0.217949	0.628341
Family Conflict	0.548511	0.394532	0.229274	0.605468
Gender	0.387539	0.179850	0.481349	0.820150
Area	0.313160	0.232052	0.282276	0.767948
∼Incomplete Family Structure	0.605050	0.199909	0.659565	0.800091
∼Parent**–**child Separation	0.700288	0.208294	0.724976	0.791706
∼Poor Parental Educational Level	0.403870	0.207122	0.421096	0.792878
∼Family Financial Hardship	0.617126	0.190470	0.714398	0.809530
∼Lack of Family Intimacy	0.526691	0.155002	0.782051	0.844998
∼Family Conflict	0.451489	0.137599	0.770726	0.862401
∼Gender	0.612461	0.243361	0.518651	0.756639
∼Area	0.686840	0.206758	0.717724	0.793242
Outcome: Attention Symptoms	Presence		Absence	
	Consistency	Coverage	Consistency	Coverage
Incomplete Family Structure	0.394185	0.194560	0.343253	0.805440
Parent–child Separation	0.297160	0.184238	0.276764	0.815762
Poor Parental Education Level	0.597194	0.178146	0.579520	0.821854
Family Financial Hardship	0.395199	0.224140	0.287751	0.775860
Lack of Family Intimacy	0.465348	0.296659	0.232071	0.703341
Family Conflict	0.538202	0.314283	0.247004	0.685717
Gender	0.407708	0.153611	0.472533	0.846389
Area	0.335700	0.201952	0.279040	0.798048
∼Incomplete Family Structure	0.605815	0.162503	0.656747	0.837497
∼Parent–child Separation	0.702840	0.169721	0.723236	0.830279
∼Poor Parental Educational Level	0.402806	0.167711	0.420480	0.832289
∼Family Financial Hardship	0.604801	0.151546	0.712249	0.848454
∼Lack of Family Intimacy	0.534652	0.127741	0.767929	0.872259
∼Family Conflict	0.461799	0.114262	0.752996	0.885738
∼Gender	0.592292	0.191068	0.527467	0.808932
∼Area	0.664300	0.162350	0.720960	0.837650

Note: Consistency indicates the degree to which cases sharing a given configuration of conditions display the outcome; values closer to 1.0 suggest a stronger association. Coverage reflects the proportion of cases with the outcome that are explained by a particular configuration; higher values indicate greater empirical relevance of the configuration. “∼” denotes the absence of condition A.

Then, we conducted crisp-set QCA analyses to examine how different configurations of the eight causal conditions contributed to each of the three outcomes—Internalizing Symptoms, Externalizing Symptoms, and Attention Symptoms—when present or absent. As shown in Table 4, the overall solution consistency was 0.842105 with coverage of 0.0138799 for Internalizing Symptoms, 0.909091 with coverage of 0.00137231 for Externalizing Symptoms, and 0.818182 with coverage of 0.0015213 for Attention Symptoms. The standard analysis generated identical complex, parsimonious, and intermediate solutions for internalizing symptoms. According to the results, internalizing symptoms present under the following configurations: (1) when Incomplete Family Structure, Poor Parental Education Level, Family Financial Hardship, Lack of Family Intimacy, Family Conflict, Female Gender, and City Area were present; (2) when Family Financial Hardship, Lack of Family Intimacy, Family Conflict, Female Gender, City Area were present AND Incomplete Family Structure, Parent–child Separation, Poor Parental Education Level were absent; (3) when Incomplete Family Structure, Parent–child Separation, Lack of Family Intimacy, Family Conflict, Female Gender, City Area were present AND Poor Parental Education Level, Family Financial Hardship were absent; OR (4) when Parent–child Separation, Poor Parental Education Level, Family Financial Hardship, Lack of Family Intimacy, Family Conflict, City Area were present AND Incomplete Family Structure, Female Gender were absent. Notably, the fourth configuration for internalizing symptoms also exhibited high consistency with the configurations leading to both externalizing symptoms and attention symptoms.

**Table 4.** **Analysis of causal condition configurations**.

	Internalizing Symptoms				Externalizing Symptoms	Attention Symptoms
	Path 1	Path 2	Path 3	Path 4		
Incomplete Family Structure	∙	∘	∙	∘	∘	∘
Parent–child Separation		∘	∙	∙	∙	∙
Poor Parental Education Level	∙	∘	∘	∙	∙	∙
Family Financial Hardship	∙	∙	∘	∙	∙	∙
Lack of Family Intimacy	∙	∙	∙	∙	∙	∙
Family Conflict	∙	∙	∙	∙	∙	∙
Female Gender	∙	∙	∙	∘	∘	∘
City Area	∙	∙	∙	∙	∙	∙
Solution Consistency	0.842105				0.909091	0.818182
Solution Coverage	0.0138799				0.00137231	0.0015213

Note: Black circles (∙) indicate the presence of a condition, and blank circles (∘) indicate its absence in the causal recipe solutions. Blank spaces represent “don’t care” conditions, meaning that the presence or absence of the condition does not influence the outcome. While four distinct configurations were found for internalizing symptoms, only one configuration was identified for both externalizing and attention symptoms.

## 4. Discussion

This study makes a novel contribution to the literature on children’s mental health by introducing a configurational perspective through the use of crisp-set QCA to examine how multiple family risk factors interact to influence mental health outcomes. While prior research has predominantly relied on variable-centered approaches—such as regression or structural equation modeling—that assume linear, additive, and independent relationships among variables, these methods often fail to capture the complex, combinatorial nature of co-occurring risks [19]. The study has employed person-centered methods, such as latent profile analysis (LPA), to identify subgroups with shared patterns of risk exposure, thus accounting for heterogeneity [21]. However, LPA does not offer insights into configurational causality. In contrast, QCA allows identification of multiple sufficient combinations of conditions leading to the same or different outcomes, accommodating asymmetrical causation and providing actionable insights for targeted interventions [18]. Specifically the crisp-set QCA results yielded three main conclusions in the present study. First, none of the hypothesized causal conditions alone were necessary or sufficient for the outcomes to occur. Second, the outcomes were more likely to occur when most of the causal conditions were present. Third, while one configuration consistently led to all three symptom dimensions, three additional configurations were uniquely linked to internalizing symptoms; in contrast, externalizing and attention symptoms shared only that single configuration, with no other distinct causal pathways identified.

Our findings have both theoretical and practical implications for understanding and addressing the cumulative effects of family risks on children’s mental health.

First, the results reinforce the CRM theory, which posits that multiple coexisting risks exert a greater impact than individual factors alone [11, 12, 13, 14]. None of the single causal conditions were necessary or sufficient for the outcomes, supporting the ecological perspective that children’s development is shaped by the interplay of various risk factors within their environments and emphasizing the importance of analyzing configurations of multiple risks rather than isolating individual predictors. One important possible explanation for the lack of significance of individual risk factors is that their effects may not be universal but rather context-dependent—that is, a specific risk may only manifest its impact in the presence (or absence) of certain other conditions. For example, financial hardship might have a stronger effect on a child’s mental health when combined with low parental education and family conflict, but may be less harmful in families with high emotional closeness or effective coping resources. This aligns with the concept of contextual moderation [40], where the influence of a particular factor is conditional upon the broader configuration of surrounding risks. The crisp-set QCA approach is well suited to capture such complexity, as it allows for the identification of these interactive, configurational effects that would otherwise be obscured in traditional analyses focusing on isolated or additive relationships.

These findings have profound implications for the Response to Intervention (RTI) practice, a widely used approach emphasizing early identification and multi-tiered intervention tailored to the child’s specific needs. RTI emphasizes early identification and intervention at varying levels of intensity, tailored to the specific needs of the child [41]. From a screening and prevention perspective, it underscores the critical need to closely monitor children exposed to multiple risks, as they are significantly more likely to develop mental health problems. Given that the likelihood of developing mental disorders increases with the accumulation of risk factors, mental health professionals need to adopt a holistic approach. For instance, traditional assessment methods should consider the cumulative effects of multiple risk factors across ecological domains, rather than simply capturing one or two isolated risks. In addition, case management systems and children’s mental health records need to incorporate a multidimensional view of the child’s environment to ensure that no aspect is overlooked, allowing for more accurate assessments and timely interventions.

From an intervention perspective, however, the results suggest that simultaneous interventions addressing all family risks may be impractical. Instead, targeting a single critical risk factor—such as family functioning or family resources—may effectively disrupt the cumulative effect of multiple risks. Addressing one pivotal stressor can initiate a positive cascade of changes, making interventions more feasible and manageable for clinicians and families alike. For example, improving family functioning—such as enhancing communication and conflict resolution skills among parents—can have a ripple effect that reduces not only the immediate emotional stress on children but also the broader impacts on behavior and social functioning. Similarly, focusing on improving family resources, such as access to education, financial support, or community services, can alleviate the strain on parents and create a more stable environment for the child. Clinicians should therefore tailor interventions to address the most pressing stressor to break the cycle of accumulated risks and to initiate a positive chain of change that supports the child’s mental health, which makes the process more manageable for clinicians and more feasible for families. By combining comprehensive monitoring with targeted intervention, this strategy provides a balanced and efficient framework for addressing mental health challenges in Chinese children.

Second, the findings align with the concepts of multifinality and equifinality, two central principles of developmental psychopathology theory. Multifinality—the phenomenon whereby the same risk factors lead to diverse outcomes [42]—is evident in our results, where an identical configuration of family risks, gender, and residential area contributed to all three symptom dimensions, reflecting a shared underlying mechanism that can lead to varied psychological manifestations. This configuration also suggests that boys are more likely to experience multiple symptom dimensions simultaneously. This finding is consistent with prior research [43, 44, 45, 46], and the co-occurrence of internalizing, externalizing, and attention problems may be explained by their shared susceptibility to adverse family environments, which contribute to emotional dysregulation, impaired executive functioning, and social difficulties [47, 48, 49, 50]. Despite the similarity in the underlying family risk configuration, outcomes may differ depending on various other factors, including the child’s temperament, resilience, coping strategies, and the presence of protective factors such as social support or positive school environments.

In contrast, equifinality—the concept that different configurations can result in the same outcome [40]—similarly supported by our identification of multiple distinct risk patterns associated with internalizing problems, particularly among girls. These results suggest that internalizing symptoms (e.g., anxious emotions and depressive moods) may arise through a broader range of pathways due to their multifactorial and often covert nature. Compared to externalizing or attention problems, internalizing symptoms are more likely to be shaped by subtle, cumulative, and subjective experiences—such as perceived emotional neglect, chronic stress, or insecure attachment—which may interact differently with individual vulnerabilities like temperament, self-concept, or emotion regulation capacity [51, 52]. This complexity allows for greater heterogeneity in the configurations leading to internalizing distress [53] and thus resulted in much higher coverage for the identified configurations compared to externalizing and attention problems. Notably, as shown in previous literature [54], girls consistently report higher levels of internalizing symptoms than boys, particularly after the age of six, a trend that intensifies during adolescence. In light of the current findings, this pattern may be partially explained by the broader range of stressors and emotional challenges girls typically encounter during this developmental period, which increases their exposure to multiple psychological risk pathways leading to internalizing problems [55].

Third, the findings of this study challenge conventional assumptions about static family risk factors. Specifically, Incomplete Family Structure, Parent–child Separation, Poor Parental Education Level, and Family Financial Hardship did not consistently emerge as risk factors across the configurations associated with mental health symptoms. In contrast, the detrimental effects of Lack of Family Intimacy and Family Conflict were consistently observed across all causal condition configurations. This observation partially aligns with the findings of Chen *et al*. [21], who found that separation from parents potentially offered a protective effect for Chinese adolescents. These results suggest that structural or demographic characteristics of families may not be inherently harmful; rather, the emotional quality of family relationships plays a more decisive and stable role in shaping children’s psychological outcomes. Accordingly, interventions aimed at promoting children’s mental health should prioritize strengthening family emotional bonds, fostering open communication, and mitigating family conflicts, rather than focusing solely on modifying family structures or addressing demographic disadvantages. Future research should also shift its focus from static family indicators to dynamic family processes, exploring how variations in emotional connectedness and conflict management contribute to different developmental trajectories.

## 5. Conclusions

Following the principles of the CRM theory and the concepts of multifinality and equifinality, this study provides novel insights into the relationships between family risk configurations and mental health symptoms in Chinese children. Using QCA, we demonstrated that mental health outcomes are influenced not by individual risk factors but by specific configurations of multiple risks. The findings highlight the importance of monitoring children exposed to cumulative family risks and suggest that targeted interventions addressing one or two key risk factors—such as family atmosphere or resources—can significantly mitigate overall mental health risks. Future research should explore these configurations longitudinally and expand the scope to include additional risk factors, providing a more comprehensive understanding of children’s developmental trajectories.

## 6. Limitations and Future Research Direction

This study has some limitations that should be addressed in future research. First, its cross-sectional design limits the ability to draw causal inferences about the relationship between family risk configurations and mental health outcomes. Future longitudinal research is needed to explore how these configurations evolve over time and their long-term impacts on children’s development. Second, the study focused on six key family risk factors, which, while representative, may not capture the full spectrum of risks affecting children. This limited scope may partially account for the relatively low coverage observed for externalizing and attention problems, which are likely influenced by a broader range of factors beyond the family context. Expanding the scope to include additional factors, such as parental mental health, community influences, and school environments, could provide a more comprehensive understanding of cumulative risks and their effects.

## Availability of Data and Materials

The data presented in this study are available upon reasonable request from the corresponding author.
